# Pre- and Apnoeic high flow oxygenation for RApid sequence intubation in The Emergency department (Pre-AeRATE): study protocol for a multicentre, randomised controlled trial

**DOI:** 10.1186/s13063-019-3305-8

**Published:** 2019-04-04

**Authors:** Mui Teng Chua, Faheem Ahmed Khan, Wei Ming Ng, Qingshu Lu, Matthew Jian Wen Low, Ying Wei Yau, Amila Punyadasa, Win Sen Kuan

**Affiliations:** 1 Emergency Medicine Department, National University Hospital, National University Health System, Level 4, 9 Lower Kent Ridge Road, Singapore, 119085 Singapore; 2 Department of Surgery, Yong Loo Lin School of Medicine, National University of Singapore, Singapore, Singapore; 3 Emergency Department, Ng Teng Fong General Hospital, Singapore, Singapore; 4 Department of Intensive Care Medicine, Ng Teng Fong General Hospital, Singapore, Singapore; 5 Singapore Clinical Research Institute (SCRI) Pte Ltd, Singapore, Singapore; 6 Department of Emergency Medicine, Lee Kong Chian School of Medicine, Nanyang Technological University, Singapore, Singapore

**Keywords:** Rapid sequence intubation, High flow nasal oxygenation, Pre-oxygenation, Apnoeic oxygenation

## Abstract

**Background:**

Maintaining adequate oxygenation during rapid sequence intubation (RSI) is imperative to prevent peri-intubation adverse events that can lead to increased duration of hospital and intensive care unit stay, or a prolonged vegetative state requiring long-term institutionalisation. Despite employing current best practices during RSI, desaturation during intubation still occurs. High-flow nasal cannula (HFNC) oxygenation may potentially improve oxygenation during pre- and apnoeic oxygenation to allow a longer safe apnoeic time for RSI.

**Objective:**

We aim to test the hypothesis that the use of humidified high-flow oxygenation via nasal cannula at 60 L/min maintains higher oxygen saturation compared with current usual care of non-rebreather mask and standard nasal cannula at an oxygen flow rate of 15 L/min for pre- and apnoeic oxygenation.

**Methods:**

This is a multi-centre randomised controlled trial enrolling adult patients aged 21 years and older who require rapid sequence intubation due to medical, surgical, or traumatic conditions in the Emergency Departments (EDs) of the National University Hospital and the Ng Teng Fong General Hospital. Eligible patients will undergo randomisation at an equal ratio into intervention or control arms. The primary endpoint will be the lowest oxygen saturation achieved during the first intubation attempt from time of administration of paralytic agent until quantitative end-tidal carbon dioxide is detected if the first intubation attempt is successful, or until the start of the second attempt if it is not.

**Discussion:**

Prolongation of safe apnoea time through maintenance of oxygen saturation above 90% using HFNC oxygenation during RSI could potentially change current clinical practice, improve standard of care, and translate to better outcomes for patients.

**Trial registration:**

ClinicalTrials.gov, NCT03396094. Registered on 10 January 2018.

**Electronic supplementary material:**

The online version of this article (10.1186/s13063-019-3305-8) contains supplementary material, which is available to authorized users.

## Introduction

Rapid sequence intubation (RSI) is the most common method of intubation used in the Emergency Department (ED) [1]. RSI involves the immediate administration of a paralytic agent following delivery of an induction agent that brings about unresponsiveness; it is a procedure which aims for successful endotracheal intubation with no ventilation via bag-valve-mask while awaiting the onset of paralysis. Under these circumstances, the maintenance of oxygen saturation during the apnoeic phase depends on the patient’s underlying oxygen reserve, and the adequacy of pre- and apnoeic oxygenation.

Critically ill patients have a shorter safe apnoeic time due to physiological stressors that accompany critical illnesses, such as decreased cardiac output, increased shunting, and reduced pulmonary reserves [2]. Oxygen saturation in these patients can rapidly drop to a critical hypoxic state of less than 70% within seconds [3]. Furthermore, with increasing prevalence of obesity both locally and worldwide [4, 5] from increasing affluence, a large proportion of our critically ill patients are also obese, which further shortens safe apnoeic times. It is estimated that a moderately ill normal-sized adult would reach an oxygen saturation (SpO_2_) of 90% at slightly under 5 min, while a 127-kg obese adult would reach such a level within 2.5 to 3 min [6].

The risk of adverse outcomes escalates with an increasing number of attempts at endotracheal intubation and with the development of hypoxia [7–9]. Prolongation of safe apnoea time and adequate pre-oxygenation would ameliorate some of these negative outcomes by preventing hypoxia and allowing more time to achieve successful intubation. As such, the current usual practice of non-rebreather mask for pre-oxygenation and standard nasal cannula of 15 L/min for apnoeic oxygenation may not be adequate in maintaining oxygenation during RSI of critically ill ED patients.

The purpose of this multi-centre randomised controlled trial is to determine the best practices for maintaining oxygenation during rapid sequence intubation of critically ill patients in the ED. Our primary aim is to test the hypothesis that the use of humidified high-flow oxygenation via nasal cannula (HFNC) at 60 L/min for pre-oxygenation and apnoeic oxygenation will maintain a higher SpO_2_ when compared with current usual care of non-rebreather mask (NRM) for pre-oxygenation and standard nasal cannula of 15 L/min of oxygen flow for apnoeic oxygenation.

## Methods

**Fig. 1:**
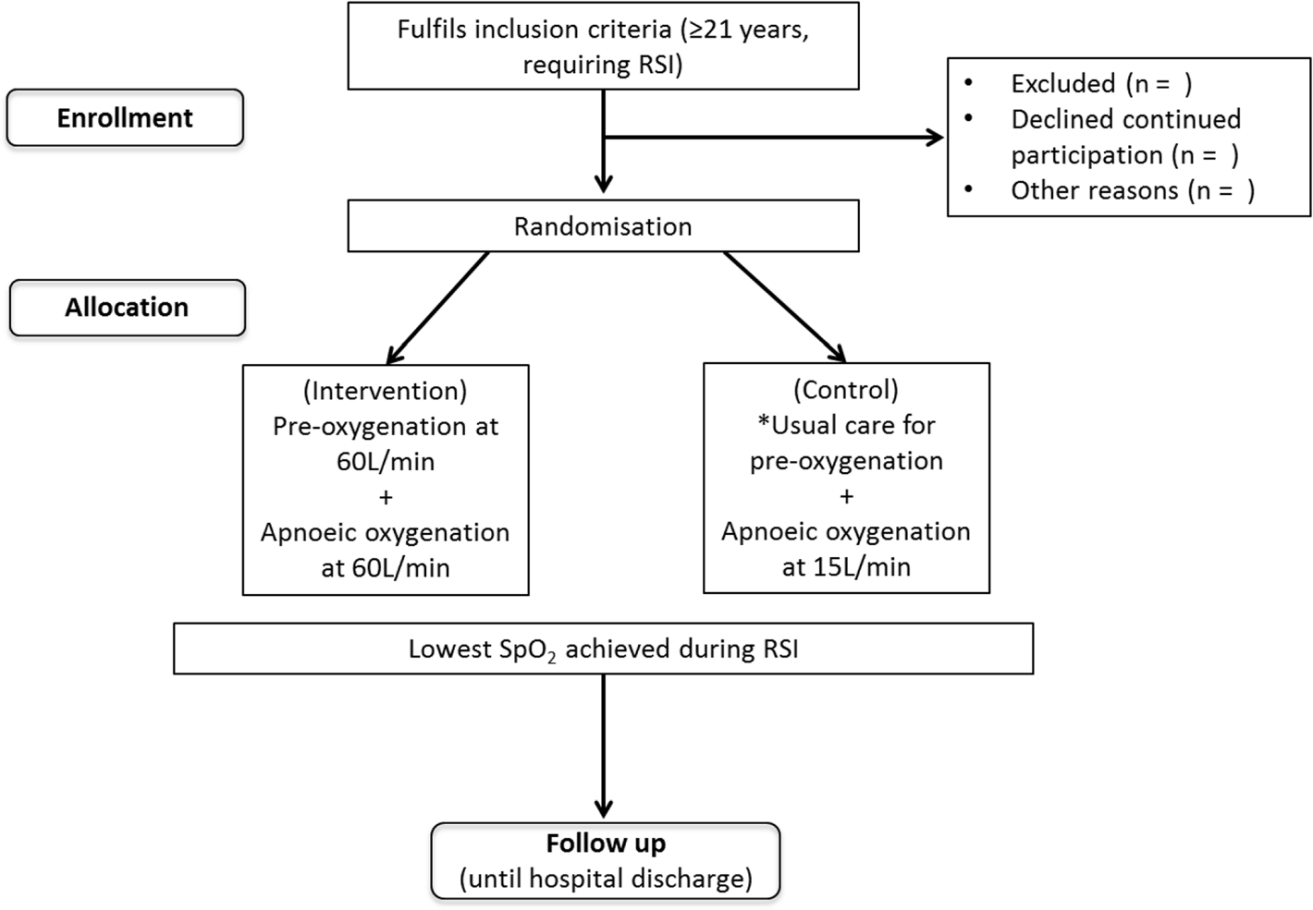
Randomisation flow chart. *Usual care for pre-oxygenation involves the use of non-rebreather mask. RSI rapid sequence intubation, SpO_2_ saturation of peripheral oxygen

**Fig. 2:**
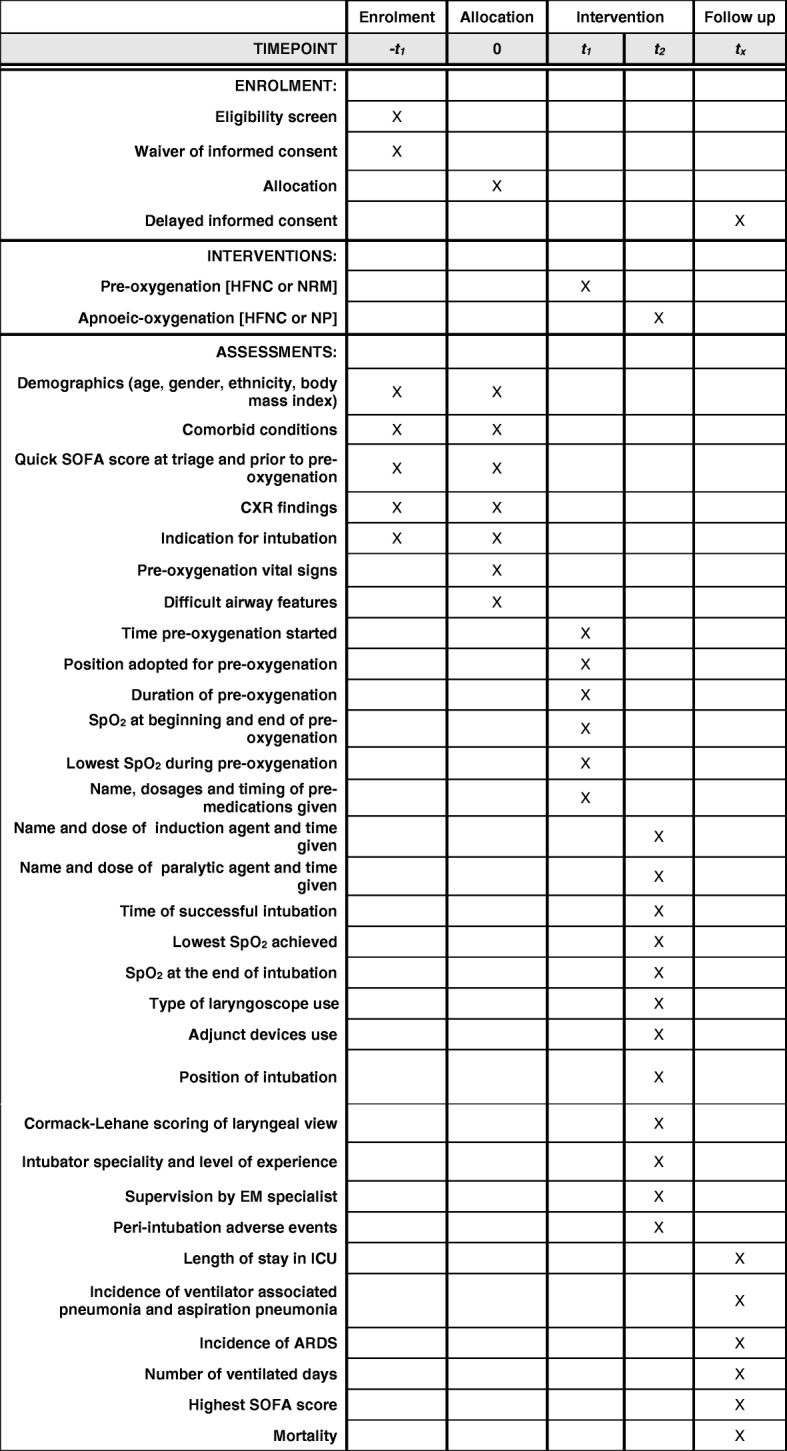
Standard Protocol Items: Recommendations for Interventional Trials (SPIRIT) diagram. ARDS acute respiratory distress syndrome, CXR chest x-ray, EM emergency medicine, HFNC high-flow nasal cannula, ICU intensive care unit, NP nasal prongs, NRM non-rebreather mask, SOFA Sequential Organ Failure Assessment, SpO_2_ saturation of peripheral oxygen

This will be a randomised controlled study (Fig. 1), enrolling adult patients aged 21 years and older who require RSI due to medical, surgical, and traumatic conditions in the EDs of the National University Hospital (NUH) and the Ng Teng Fong General Hospital (NTFGH), Singapore. The planned Consolidated Standards of Reporting Trials (CONSORT) flow diagram is presented in Fig. 1. The protocol is reported according to the Standard Protocol Items: Recommendations for Interventional Trials (SPIRIT; Fig. 2 and Additional file 1).

The NUH is a 1225-bed tertiary academic medical centre in Singapore and its ED receives over 110,000 attendances annually, of which about 47% of the cases require urgent (42.5%) or immediate (4.5%) care. The NTFGH is a 700-bed acute care general hospital located within the same healthcare cluster with annual ED attendances that approximate over 120,000. Both institutions perform an average of 12 to 20 RSIs per month.

### Recruitment

Research assistants will be stationed in both EDs to screen, enrol, and randomise eligible patients during office hours on weekdays. After office hours, attending physicians with eligible patients will call the study investigators for off-site randomisation around the clock. Prior to study commencement, all nurses and clinicians working in both EDs have been trained on the use of HFNC oxygenation, the study protocol, and data collection using paper-based case report forms. In the control arm, the use of NRM for pre-oxygenation and standard nasal cannula of 15 L/min of oxygen flow for apnoeic oxygenation is current standard clinical practice. During the protocol training for this study, this routine practice is further reinforced for compliance.

### Ethics and consent

Ethics approval for waiver of consent at the time of enrolment and delayed informed consent was obtained from the National Healthcare Group’s (NHG) Domain Specific Review Board (DSRB reference number 2017/00348) in accordance with regulations for clinical trials under emergency situations [10].

Patients who are hypoxic and critically ill will be cognitively impaired and, therefore, will not have the appropriate mental capacity to comprehend and retain information to provide consent. Additionally, RSI is a time-sensitive intervention and consent from the legally acceptable representative (LAR) is not practically plausible for the following reasons: 1) the next-of-kin may not always be around at the initial stages of attendance in the ED; 2) contact details of the patient’s next-of-kin is not always available; and 3) immediate evaluation and treatment of the patient should not be delayed. If an appropriate LAR is present, the opportunity to reject study enrolment will be offered.

Declaration for waiver of consent at time of enrolment will be certified by a study investigator and an independent emergency medicine specialist. Consent for continued participation will be obtained from the participant or LAR at the earliest possible subsequent time by the research assistants or study investigators.

### Randomisation

Subjects will be randomised at an equal ratio into two treatment combinations (Fig. 1). Random blocks of variable sizes will be selected via a web-based randomisation service. The block lengths will be kept unknown to the site as per ICH E9 guidelines. However, block randomisation will ensure the numbers in each group will remain similar throughout the study.

Allocation concealment will be maintained until registration and the randomisation process is completed. This is achieved through web-based randomisation which only reveals the allocation at the time of enrolment. However, due to the nature of the intervention it will not be possible to blind the treating clinician, ED staff members, or patient after allocation has occurred. As the outcome measures in our study are objective physiological measurements, the risk of bias remains low [11]. Clinicians in the admitting intensive care unit (ICU) will be blinded to the patients’ allocation to any of the study arms as allocation to either arm will not be documented in the patient’s electronic medical records.

### Inclusion criteria

Adult patients aged 21 years and older who require RSI due to medical, surgical, and traumatic conditions in the Emergency Departments of the National University Hospital and the Ng Teng Fong General Hospital will be eligible for study enrolment.

### Exclusion criteria

Patients who fulfil any of the following criteria will be excluded:

- Patients with “do-not-resuscitate” orders
- Crash, awake, or delayed sequence intubations
- Patients requiring non-invasive positive pressure ventilation
- Cardiac arrest
- Clinical suspicion or confirmed diagnosis of base of skull fractures or severe facial trauma that precludes nasal cannula placement
- Vulnerable patient populations (e.g. pregnant women, prisoners)

### Treatment protocol

In the intervention arm using HFNC, the patients will receive oxygenation with 60 L/min of warm and humidified oxygen at 37 °C and a fraction of inspired oxygen (FiO_2_) of more than 0.9 using the AIRVO™ 2 Humidifier with Integrated Flow Generator (Fisher & Paykel Healthcare, Auckland, New Zealand) during the pre- and apnoeic oxygenation phases.

The control group will be managed in accordance with current best practice by performing pre-oxygenation using NRM that has FiO_2_ of 0.6 to 0.8 [12] and given 15 L/min of non-humidified and non-heated oxygen via nasal cannula.

Both groups will receive at least 3 min of pre-oxygenation [3, 13, 14]. Premedication (if required) will be given at the start of pre-oxygenation [15]. Once 3 min or more of pre-oxygenation is completed, induction medications are administered and apnoeic oxygenation commenced as per the assigned treatment group. Intubation attempts start after 30 to 45 s (onset time of paralytic agent, typically succinylcholine or rocuronium). If the intubator is of resident grade, only two attempts are allowed before escalating to an EM specialist. Each attempt is defined as the passage of the laryngoscope through the mouth. The end of intubation is defined as correct placement of endotracheal tube with confirmation using quantitative end-tidal carbon dioxide (ETCO_2_).

### Study endpoints

The primary endpoint to be measured is the lowest SpO_2_ achieved during the first intubation attempt. This is defined as the time taken from administration of paralytic agent until quantitative ETCO_2_ is detected post-intubation if it is successful, or until the start of the second intubation attempt for failed intubations. The first intubation attempt is defined as the first attempt to insert an endotracheal tube into the oropharynx, and SpO_2_ will be measured using the pulse oximeters Philips Intellivue MP30 Patient Monitor and Zoll R Series® defibrillator in two different areas. Continuous SpO_2_ monitoring with waveform tracing will be carried out throughout the intubation process with the SpO_2_ values before pre-oxygenation, at the end of pre-oxygenation (which is the start of induction), and at the end of intubation being recorded with a timestamp on the case report forms. Any SpO_2_ readings below 100% during the pre-oxygenation and intubation phases will also be recorded. Nurses and clinicians in the departments have been trained to only record SpO_2_ readings with adequate waveforms. Two monitors will be used to mitigate any suboptimal detection of SpO_2_ tracing.

All this information is documented real-time on paper-based case report forms by a trained scribe nurse who observes the entire process at the foot of the patient’s trolley but is not involved in the intubation process. The time taken is recorded based on a wall-mounted digital clock and the number of intubation attempts determined by the scribe nurse and intubating clinician. Any discrepant recording between the scribe nurse and intubating clinician will be adjudicated by another clinician in the resuscitation team.

The secondary endpoints evaluated are as follows:

- Number of attempts at intubation
- Safe apnoea time during intubation
- Incidence of SpO_2_ < 90%
- Names and dosages of induction agents used
- Peri-intubation adverse events such as hypotension, hypertension, tachycardia, bradycardia, regurgitation, aspiration, cardiac arrhythmia, cardiac arrest during RSI, oropharynx or dental trauma
- Length of time to successful intubation
- Incidence of ventilator-associated pneumonia and aspiration pneumonia (by radiological and clinical diagnoses)
- Number of ventilated days
- In-hospital mortality in ICU and on discharge
- Highest Sequential Organ Failure Assessment (SOFA) score
- Incidence of acute respiratory distress syndrome (ARDS)

Other data to be collected include the baseline demographic characteristics, vital signs, difficult airway features, induction, and paralytic medications given and their timing of administration (Table 1).

**Table 1:** Clinical data collected (with no identifying data)

(a) Pre-oxygenation	• Time pre-oxygenation started • Position adopted for pre-oxygenation • Duration of pre-oxygenation (time of induction = end of pre-oxygenation) • SpO_2_ at beginning and end of pre-oxygenation (i.e. prior to induction) • Mode of pre-oxygenation (HFNC, NRM) • Lowest SpO_2_ during pre-oxygenation • Name, dosages, and timing of pre-medications given
(b) Intubation	• Time induction agent given, name of induction agent, and dose • Time paralytic agent given, name of paralytic agent, and dose • Time of successful intubation • Lowest SpO_2_ achieved during subsequent intubation attempts (if unsuccessful at first attempt) • SpO_2_ at the end of intubation • Type of laryngoscope use (direct/articulating/video) • Adjunct devices use (e.g. bougie) • Position of intubation • Cormack-Lehane scoring of laryngeal view (state direct or video view) • Intubator speciality and level of experience (by post-graduate year) • Supervision by EM specialist (yes/no) • Peri-intubation adverse events: hypotension, hypertension, tachycardia, bradycardia, regurgitation, aspiration, cardiac arrhythmia, cardiac arrest during RSI, oropharynx or dental trauma
(c) Clinical outcomes	• Length of stay in ICU • As in secondary endpoints
(d) Difficult airway features	• Reduced neck mobility or on cervical collar • Mallampati score • Mouth opening • Thyromental distance • Airway obstruction • Facial trauma (if not severe enough to preclude recruitment as per exclusion criteria) • Blood or vomitus in airway
(e) Others	• Demographics (age, gender, ethnicity, body mass index) • Comorbid conditions (congestive cardiac failure, ischemic heart disease, atrial fibrillation, chronic renal disease, chronic obstructive pulmonary disease, asthma, obstructive sleep apnoea, diabetes, previous head and neck radiation, head and neck mass or infection, upper gastrointestinal bleeding) • Pre-oxygenation vital signs (temperature, heart rate, systolic BP, diastolic BP, mean arterial pressure, Glasgow Coma Scale) • Quick SOFA score at triage and prior to pre-oxygenation • CXR findings • Indication for intubation • Vasopressor use

*BP* blood pressure, *CXR* chest x-ray, *EM* emergency medicine, *HFNC* high-flow nasal cannula, *ICU* intensive care unit, *NRM* non-rebreather mask, *RSI* rapid sequence intubation, *SOFA* Sequential Organ Failure Assessment, *SpO*_*2*_ saturation of peripheral oxygen

Only the index ED visit is included in the study. There will be no repeat visits. Patients’ clinical outcomes as per secondary endpoints will be followed up electronically using the institutions’ electronic medical record systems.

### Sample size calculation

Based on our preliminary data and a previous study [16], we anticipate a standard deviation of 14% in the lowest SpO_2_. Enrolment of 184 patients (92 patients in each of the control and intervention groups) will provide statistical power of 80% (with a two-sided α of 0.05) to detect a 6% difference in lowest SpO_2_ [17], allowing for a 5% dropout.

### General approach for statistical analysis

The primary analysis will be an intention-to-treat analysis, comparing the primary outcome of lowest SpO_2_ achieved between the two groups. For a per-protocol analysis, patients who used an oxygenation device different from the allocated arm will be excluded. Descriptive statistics of lowest SpO_2_ (e.g. mean, standard deviation, median, and interquartile range) will be reported for each group. Comparison of mean (or median) lowest SpO_2_ between groups will be made using two-sample *t* test or non-parametric test as appropriate. The 95% confidence intervals of the difference in lowest SpO_2_ will be provided. Categorical data will be compared using the Fisher’s exact test. Linear and logistic regression will be performed to identify predictors for lowest SpO_2_ and hypoxia, respectively; variables to be evaluated include variables with a *p* value < 0.10 and clinically relevant variables, namely clinical indication for intubation, difficult airway features, body mass index, duration of pre-oxygenation, time taken for intubation, number of intubation attempts, intubator specialty, and level of training.

Subgroup analyses will be performed for subgroups of interest, including recruiting hospital, indication for intubation, and the presence of at least one difficult airway feature. For the primary endpoint of lowest SpO_2_ and the secondary endpoints of number of attempts at intubation, incidence of SpO_2_ less than 90%, and incidence of peri-intubation, adverse events will be reported as subgroups.

All data analysis will be performed with STATA version 14.0 software (StataCorp, College Station, TX, USA). *P* values < 0.05 will be considered statistically significant.

### Data handling

Paper-based case report forms for data collection will contain only trial numbers and no patient identifiers to maintain confidentiality. Data entry will also be double-checked independently by two investigators. These forms will be kept in a locked cabinet in the EDs of the NUH and the NTFGH with access granted to study investigators only. An enrolled participant list linking trial numbers and personal information will be stored separately in a password-protected file. Electronic data will be recorded in the Research Electronic Data Capture (REDCap) system and maintained at the Singapore Clinical Research Institute’s (SCRI) secured server. Authorised personnel will be assigned user IDs and passwords to gain access to the database. Electronic data entered will be systematically checked by built-in edit checks.

Authors of the final trial manuscript will make significant contributions to the design, conduct, interpretation, and reporting of this trial. Our trial results will be presented in conferences and journal publications. The funder of this trial and involved clinicians of both hospitals will be provided with a summary of the results after data analysis is completed.

### Quality assurance and monitoring

Quality assurance and trial monitoring will be performed by an independent data monitoring and safety team based in each of the institutions. Quarterly reviews will be performed to ensure adherence to the study protocol and the integrity of data collection. Serious adverse events related to the study interventions will be reported to the NHG DSRB, the local institutional review board. All medical expenses incurred for the treatment of physical injuries due to the trial procedure given under the plan for this study will be compensated by the respective hospitals.

## Discussion

Adequate pre-oxygenation is the cornerstone of successful RSI and an essential step in achieving prolonged safe apnoeic times during intubation [2]. Pre-oxygenation with a high FiO_2_ de-nitrogenates the lungs and maximises oxygen reserve and oxygen saturation in the bloodstream [3]. The standard of care is to apply a non-rebreather mask (NRM) with tidal volume breathing for 3 min for pre-oxygenation. However, depending on the patient’s respiratory rate and depth of breathing which constitutes their minute volume, the actual FiO_2_ delivered may vary from 0.8 to 0.6 [12], sometimes even lower. Other more efficacious methods such as applying positive airway pressure and pre-oxygenating in a head-up position of 20° have been described, but are not always possible in emergency situations or with obtunded patients [18, 19].

HFNC oxygenation has been gaining interest as an alternative method of pre-oxygenation and apnoeic oxygenation [20]. It can deliver up to 60 L/min of oxygen and an FiO_2_ of 1.0. As it is humidified and heated, the high flow is well tolerated by awake patients [21]. Moreover, the high flow has been shown to wash out carbon dioxide in anatomical dead space, create positive airway pressure adequate enough for alveolar recruitment, and maintain a constant FiO_2_, regardless of the patient’s minute volume [20]. Additionally, it has the convenience of continuing oxygenation during the apnoeic phase after induction and paralysis, since the high flow maintains a positive end-expiratory pressure and can insufflate the lungs, resulting in apnoeic oxygenation. Apnoeic oxygenation is possible due to the differential solubility of oxygen and carbon dioxide in blood. Carbon dioxide is more soluble and therefore moves less readily down its concentration gradient from the bloodstream into the alveoli during apnoea. As a result, more oxygen moves from the alveoli into the bloodstream. This creates a sub-atmospheric pressure in the alveoli, allowing oxygen to flow from the pharynx to the alveoli during apnoea.

Despite this sensible physiological rationale, a recent large randomised controlled trial reported no difference in median lowest arterial oxygen saturation in ICU patients with apnoeic oxygenation using 15 L/min of nasal cannula compared with no apnoeic oxygenation at all [22]. However, limitations in this study prevent definitive conclusions to be drawn. Firstly, the oxygen flow of 15 L/min was delivered through routine nasal cannulae, which are not designed for such flows. Secondly, a flow rate of 15 L/min may not be high enough to provide apnoeic oxygenation; it has been shown that flows of 30 L/min are necessary to generate positive airway pressures [23]. Thirdly, the cohort in the study consisted of mostly medical ICU patients with advanced respiratory failure, in whom 15 L/min of oxygenation may not generate enough positive airway pressure to overcome pulmonary shunting. Furthermore, the results cannot be generalised to patients who do not typically present to medical ICUs, for instance patients with severe trauma or a surgical diagnosis. There have been conflicting results from previous studies evaluating pre-oxygenation and apnoeic oxygenation using true HFNC. The PREOXYFLOW trial is the largest randomised controlled trial to date (*n* = 124) and found no difference in the lowest SpO_2_ achieved when HFNC oxygenation was compared with a high fraction-inspired oxygen facial mask for pre-oxygenation. These results differ from another quasi-experimental before-after study (*n* = 101), which showed that patients who were pre-oxygenated with HFNC had significantly higher SpO_2_ during the apnoeic phase [17]. Both studies were conducted in ICU patients and continued the HFNC during the apnoeic phase for apnoeic oxygenation.

Until now, there have been no studies conducted in the ED investigating the use of HFNC for pre- and apnoeic oxygenation. The incidence of failed intubations in the emergency setting is estimated to be 20 times that compared with intubation done electively [24], and ED patients differ from ICU patients because they are less likely to have been fasted thus increasing the risk of aspiration if re-oxygenation with bag valve mask is required during the intubation attempts. Aspiration of gastric contents contributes to increased risk of death during intubation [25]. Furthermore, hypoxia from failure to intubate is associated with increased ICU length of stay, irreversible brain damage, increased number of ventilated days, and higher incidence of tracheostomy [26]. This translates to longer hospital stays, adversely affecting overall bed occupancy, as well as higher healthcare costs. Given the existing high occupancy in Singapore’s restructured hospitals, which is expected to worsen as the population ages, it is vital that we prevent such adverse events from occurring by maintaining the lowest SpO_2_ above 90% and prolonging safe apnoea times for intubation without the need for bag valve mask ventilation for re-oxygenation during RSI. If our hypothesis is confirmed, the results from this study could potentially change current clinical practice and reduce demands on an already stressed healthcare system.

### Limitations

Our study has potential limitations. Firstly, by using the study intervention for both pre- and apnoeic oxygenation, it would not be possible to attribute any favourable results of HFNC to either process. However, this would not have made any clinical significance or impact in a pragmatic situation. The switch from pre- to apnoeic oxygenation is so rapid and takes seconds, and thus clinicians would appreciate a seamless process and intervention using HFNC for both types of oxygenation.

Second, in order to limit the variation of care under the control arm, we have excluded patients who required non-invasive positive pressure ventilation. This may make our results and conclusions not generalizable to this group of patients.

## Trial status

Recruitment for the Pre-AeRATE trial is currently ongoing and expected to be completed by November 2019. This study protocol reports protocol version 2.0, dated 29 December 2017.

## Additional file

Additional file 1: SPIRIT checklist. (DOCX 61 kb)

## References

[1] Walls RM, Brown CA, Bair AE (2011). Emergency airway management: a multi-center report of 8937 Emergency Department intubations. J Emerg Med.

[2] Thorpe CM, Gauntlett IS (1990). Arterial oxygen saturation during induction of anaesthesia. Anaesthesia.

[3] Weingart SD, Levitan RM (2012). Preoxygenation and prevention of desaturation during emergency airway management. Ann Emerg Med.

[4] Goh LG, Pang J (2012). Obesity in Singapore, prevention and control. SFP.

[5] Wyatt SB, Winters KP, Dubbert PM (2006). Overweight and obesity: prevalence, consequences, and causes of a growing public health problem. Am J Med Sci.

[6] Benumof JL, Dagg R, Benumof R (1997). Critical hemoglobin desaturation will occur before return to unparalyzed state from 1 mg/kg succinylcholine. Anesthesiology.

[7] Sakles JC, Chiu S, Mosier J (2013). The importance of first pass success when performing orotracheal intubation in the emergency department. Acad Emerg Med.

[8] Hasegawa K, Shigemitsu K, Hagiwara Y (2012). Association between repeated intubation attempts and adverse events in emergency departments: an analysis of a multicenter prospective observational study. Ann Emerg Med.

[9] Mort TC (2004). Emergency tracheal intubation: complications associated with repeated laryngoscopic attempts. Anesth Analg.

[10] Munn MW. The legislative framework governing clinical trials in Singapore. http://www.asiabiotech.com/10/1021/1210_1215.pdf. Accessed 23 May 2018.

[11] Wood L, Egger M, Jüni P (2008). Empirical evidence of bias in treatment effect estimates in controlled trials with different interventions and outcomes: meta-epidemiological study. BMJ.

[12] Pruitt W, Jacobs M (2003). Breathing lessons: basics of oxygen therapy. Nursing.

[13] Baraka AS, Taha SK, Aouad MT (1999). Preoxygenation: comparison of maximal breathing and tidal volume breathing techniques. Anaesthesiology.

[14] Hamilton WK, Eastwood DW (1955). A study of denitrogenation with some inhalation anesthetic systems. Anaesthesiology.

[15] Walls RM, Murphy MF. Manual of emergency airway management. 4th ed. Philadelphia: Lippincott Williams & Wilkins; 2012.

[16] Vourc’h M, Asfar P, Volteau C, et al. High-flow nasal cannula oxygen during endotracheal intubation in hypoxemic patients: a randomized controlled clinical trial. Intensive Care Med. 2015;41:1538–48. 10.1007/s00134-015-3796-z 25869405

[17] Miguel-Montanes R, Hajage D, Messika J (2015). Use of high-flow nasal cannula oxygen therapy to prevent desaturation during tracheal intubation of intensive care patients with mild-to-moderate hypoxemia. Crit Care Med.

[18] Baillard C, Fosse J-P, Sebbane M (2006). Noninvasive ventilation improves preoxygenation before intubation of hypoxic patients. Am J Respir Crit Care Med.

[19] Ramkumar V, Philip FA (2011). Preoxygenation with 20 degrees head-up tilt provides longer duration of non-hypoxic apnea than conventional preoxygenation in non-obese healthy adults. J Anesth.

[20] Nishimura M. High-flow nasal cannula oxygen therapy in adults. J Intensive Care. 2015;3:15. 10.1186/s40560-015-0084-5 PMC4393594 25866645

[21] Parke RL, McGuinness SP, Eccleston ML (2011). A preliminary randomized controlled trial to assess effectiveness of nasal high-flow oxygen in intensive care patients. Respir Care.

[22] Semler MW, Janz DR, Lentz RJ (2016). Randomized trial of apneic oxygenation during endotracheal intubation of the critically ill. Am J Respir Crit Care Med.

[23] Ritchie JE, Williams AB, Hockey H (2011). Evaluation of a humidified nasal high flow oxygen system, using oxygraphy, capnography, and measurement of upper airway pressures. Anaesth Intensive Care.

[24] Cook TM, MacDougall-Davis SR (2012). Complications and failure of airway management. Br J Anaesth.

[25] Woodall N, Frerk C, Cook TM (2011). Can we make airway management (even) safer? Lessons from national audit. Anaesthesia.

[26] Cook TM, Benger J (2011). Major complications of airway management in the UK: results of the Fourth National Audit Project of the Royal College of Anaesthetists and the Difficult Airway Society. Part 2: intensive care and emergency departments. Br J Anaesth.

